# Comparison of methods to engage diverse stakeholder populations in prioritizing PrEP implementation strategies for testing in resource-limited settings: a cross-sectional study

**DOI:** 10.1186/s43058-023-00457-9

**Published:** 2023-07-12

**Authors:** Sarah Hicks, Felix Abuna, Ben Odhiambo, Julia C. Dettinger, Nancy Ngumbau, Laurén Gómez, Joseph Sila, George Oketch, Enock Sifuna, Bryan J. Weiner, Grace C. John-Stewart, John Kinuthia, Anjuli D. Wagner

**Affiliations:** 1 Department of Epidemiology, University of Washington, Seattle, WA USA; 2 Department of Medicine, University of Washington, Seattle, WA USA; 3 Kenyatta National Hospital, Nairobi, Kenya; 4 Department of Global Health, University of Washington, Seattle, WA USA; 5 Department of Health Systems and Population Health, University of Washington, Seattle, WA USA; 6 Departments of Pediatrics, University of Washington, Seattle, WA USA

**Keywords:** Implementation strategy, Implementation determinants, Prioritization, Nominal group technique

## Abstract

**Background:**

There is a lack of consensus about how to prioritize potential implementation strategies for HIV pre-exposure prophylaxis (PrEP) delivery. We compared several prioritization methods for their agreement and pragmatism in practice in a resource-limited setting.

**Methods:**

We engaged diverse stakeholders with clinical PrEP delivery and PrEP decision-making experience across 55 facilities in Kenya to prioritize 16 PrEP delivery strategies. We compared four strategy prioritization methods: (1) “past experience surveys” with experienced practitioners reflecting on implementation experience (*N* = 182); (2 and 3) “pre- and post-small-group ranking” surveys before and after group discussion (*N* = 44 and 40); (4) “go-zone” quadrant plots of perceived effectiveness vs feasibility. Kendall’s correlation analysis was used to compare strategy prioritization using the four methods. Additionally, participants were requested to group strategies into three bundles with up to four strategies/bundle by phone and online survey.

**Results:**

The strategy ranking correlation was strongest between the pre- and post-small-group rankings (Tau: 0.648; *p* < 0.001). There was moderate correlation between go-zone plots and post-small-group rankings (Tau: 0.363; *p* = 0.079) and between past-experience surveys and post-small-group rankings (Tau: 0.385; *p* = 0.062). For strategy bundling, participants primarily chose bundles of strategies in the order in which they were listed, reflecting option ordering bias. Neither the phone nor online approach was effective in selecting strategy bundles. Participants agreed that the strategy ranking activities conducted during the workshop were useful in prioritizing a final set of strategies.

**Conclusions:**

Both experienced and inexperienced stakeholder participants’ strategy rankings tended to prioritize strategies perceived as feasible. Small group discussions focused on feasibility and effectiveness revealed moderately different priorities than individual rankings. The strategy bundling approach, though less time- and resource-intensive, was not effective. Future research should further compare the relative effectiveness and pragmatism of methodologies to prioritize implementation strategies.

**Supplementary Information:**

The online version contains supplementary material available at 10.1186/s43058-023-00457-9.

Contributions to the literature

- Comparative evidence on methods to prioritize implementation strategies to address determinants is limited but essential to advance practical applications of implementation science globally.
- We compared four strategy prioritization methods and two strategy bundling exercises for use by diverse stakeholders.
- Stakeholders tended to prioritize strategies that were familiar and perceived as more feasible over strategies perceived as more effective.
- The strategy bundling approach tested a less time- and resource-intensive method but was not effective.
- The strategy prioritization activities utilized during the workshop were acceptable to participants.

## Introduction

While there are several established methods for barrier-strategy matching, methods for prioritizing which implementation strategies to test or deliver remains a considerable challenge in implementation science (IS), particularly prioritizing strategies through engaging a diverse set of stakeholders. Stakeholder engagement has been shown to increase acceptability of proposed interventions, enhance community buy-in, and ensure cultural appropriateness of the intervention [1–3]. Stakeholder-driven implementation science methods—including prioritization efforts—are a critical frontier in implementation science. Some of the posited advantages of stakeholder-driven approaches are that specification of strategies can be more specific due to their content and context expertise and de-prioritization of strategies that may be theoretically well-suited to barriers but not feasible within the given context. Many of the existing methods for mapping and criteria for selecting strategies are “top down” approaches, in which scientific experts decide what to implement in consultation with community or content experts. In this paper, we present methods that are “bottom up,” which allow diverse stakeholders to directly prioritize strategies to test based on their own firsthand experiences and understanding, without extensive implementation science training.

In the process of choosing which implementation strategies to test or deliver, there are several robust methods for barrier-strategy mapping or matching that include stakeholder engagement. Intervention mapping—which has been applied to IS in implementation mapping focuses on highly impactful barriers identified during planning that are mapped to appropriate strategies using a theoretical understanding of the relationships between barriers and strategies [4–6]. The Behavior Change Wheel and COM-B model are also well-suited to barrier-strategy matching, particularly through highlighting the behavioral mechanism through which a strategy acts. The CFIR-ERIC matching tool is well-suited to provide a provisional list of ERIC strategies that are matched to CFIR-derived barriers. However, neither implementation mapping, nor BCW-COM-B, nor CFIR-ERIC matching tool have scales or criteria for prioritization built in. The study that yielded the creation of the ERIC and linked publications of groupings of similar strategy types did utilize feasibility and effectiveness as criteria, but this tool does not include this type of exercise for prioritization after candidate strategy selection. Therefore, despite barrier-strategy matching tools existing to guide matching, we are still left with the problem of how we prioritize among candidate strategies that come out of a matching exercise. APEASE provides a series of criteria that can be used to inform prioritization efforts but no specific approach for how to translate the 6 criteria into a ranked list. APEASE is typically utilized by a content expert team with some consultation of frontline stakeholders but is not a stakeholder-driven, or bottom-up, approach. While the criteria in APEASE exist and are likely useful for driving prioritization, we are not aware of any evidence base that suggests their superiority over any other set of criteria or approach in terms of selecting ultimately impactful strategies.

Group consensus-generating tools are important and have been used variably in low-resource contexts. The Delphi method, nominal group technique, and the consensus development conference center stakeholder expertise for prioritizing problems and solutions during group discussions [1, 7–10]. Creation of “go-zone” plots—in which two selection criteria are plotted on *x*- and *y*-axes and the quadrant that is “high” for both criteria is the “go-zone”—is a stakeholder-engaged visual method of strategy prioritization [11]. Finally, a variety of ranking approaches (best–worst choice, ranking attributes, constant sum scaling, conjoint analysis, discrete choice experiment, etc.) may be used to individually rank strategies for prioritization with group averages used in lieu of consensus [12–17]. In addition to selecting which strategies to prioritize, a linked challenge is which strategies to bundle together. To guide strategy bundling, concept mapping is well-suited, revealing relationships between ideas that are explicitly mapped out based on compiled individual sorting data; these data can be used to understand which strategies might be conceptually similar, as well as important [18–20].

The existing methods of strategy prioritization described above have varying time and resource complexity profiles and researchers have called for increased comparative research and economic evaluations to compare the various methodologic approaches [21]. Additionally, there is a notable lack of comparative data about the use of these methods in low- and middle-income country (LMIC) settings; previous work has highlighted the need for comparative testing of different strategy prioritization methods to determine stakeholder feasibility and acceptability, the diversity of prioritized strategies by each method, and the efficiency of prioritization methods [6].

In this study, we sought to address the gap in comparative testing of different strategy prioritization methods in a LMIC setting. We utilized a variety of strategy ranking approaches to determine whether more or less time- and resource-intensive methods could provide similar prioritization profiles. We also aimed to provide a nuanced description of the similarities and differences in ranking outcomes between methods.

## Methods

### Study context

Pre-exposure prophylaxis (PrEP) is an evidence-based, once-daily pill that that has demonstrated efficacy and safety for use by women during pregnancy [22–24]. While PrEP delivery has been broadly scaled up for sero-discordant couples and adolescent girls and young women, PrEP delivery in MCH clinics has been slower due to the additional time required for diffusion and uptake of new service delivery methods [25–28]. Several studies in Kenya have demonstrated that integrating delivery of PrEP into maternal and child health clinics (MCH) is feasible [29–31], but barriers to successful implementation were noted. The ongoing *PrEP in Pregnancy, Accelerating Reach and Efficiency* study (PrEPARE; NCT04712994) utilized stakeholder engagement to obtain both qualitative and quantitative data regarding determinants of PrEP implementation and the prioritization of PrEP implementation strategies in MCH clinics in Kenya.

### Study design and participants

**Fig. 1:**
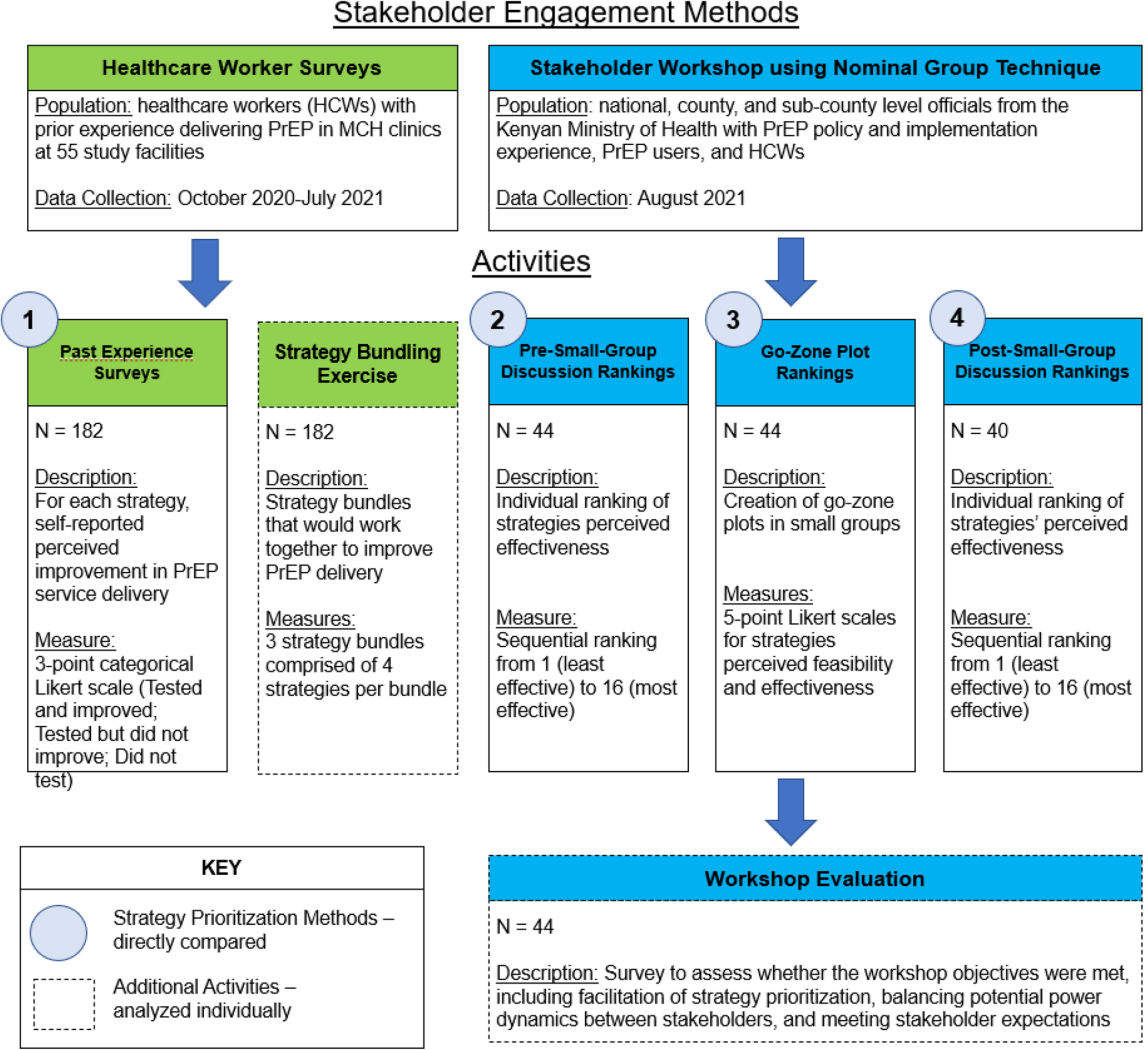
Outline of stakeholder engagement methods and prioritization activities

This was a cross-sectional study to evaluate four strategy prioritization activities and a strategy bundling exercise via two stakeholder engagement methods (Fig. 1). The data for this study was collected with diverse stakeholders in Kisumu, Homa Bay, and Siaya Counties in Kenya. Two distinct populations contributed to the sequential data collection in this study. First, healthcare workers (HCWs), who were selected for their prior experience delivering PrEP in MCH clinics at 55 facilities, participated in HCW surveys between October 2020 and July 2021. The facilities from which HCWs were recruited had previously participated in studies seeking to integrate PrEP in MCH clinics. Second, a group of diverse PrEP stakeholders were recruited for an in-person stakeholder workshop held in August 2021. Stakeholders included national- and county-level officials from the Kenyan Ministry of Health with PrEP policy and implementation experience, PrEP users, HCWs; participants were purposively sampled for their breadth of experience (e.g., government, facility, lived experience) and different levels of government service (subcounty, county, and national).

### Data collection

#### Overview

Study participants rated and ranked 16 unique PrEP delivery implementation strategies which were identified in previous qualitative work using the Consolidate Framework for Implementation Research (CFIR) to inform the question guide and deductive coding [32, 33]. Participants were initially asked about perceived benefits and challenges of PrEP implementation in MCH clinics. The main barriers identified were insufficient providers and inadequate training, insufficient space to deliver PrEP, and high volume of patients [32]. The 16 identified strategies to overcome these barriers are provided in Additional file 1. While no framework was utilized to inform strategy-barrier mapping, the selected strategies mapped reasonably well to several ERIC strategies post hoc, including revision of professional roles (task shifting), developing and distributing educational materials and conducting educational meetings (communication aids and health talks), and changing service sites (delivering PrEP and health talks in MCH) [34].

Data collection for each of the four prioritization activities and the strategy bundling exercise are summarized below. Participants also completed a brief workshop evaluation survey to assess whether the workshop objectives were met, including facilitation of strategy prioritization, balancing potential power dynamics between stakeholders, and meeting stakeholder expectations. All data was collected through online REDCap and Poll Everywhere surveys; a summary is included in Fig. 1 [35–37].

#### Strategy prioritization activities

The four prioritization activities to be compared in this paper include the following: (1) “past experience surveys” with experienced practitioners reflecting on implementation success experience; (2 and 3) ‘pre- and post-small group ranking” surveys before and after small group discussion; (4) “go-zone” quadrant plots of perceived effectiveness vs feasibility (Fig. 1). Three bundles of strategies were ultimately selected for testing: bundle 1, fast-tracking PrEP clients in MCH and retraining providers; bundle 2, task-shifting PrEP risk counseling to HIV testing service providers and training different providers to deliver PrEP; and bundle 3, PrEP health talks in waiting bays and provision of communication aids. All bundles will also include delivering PrEP in MCH clinics, retraining providers, and audit and feedback processes. Given the exploratory nature of identifying potential PrEP implementation strategies, we sought to foster community-academic partnerships across a range of stakeholder experiences (government, facility, lived experience), and we selected relatively simple prioritization activities (e.g., surveys) to facilitate non-researcher stakeholder engagement. Furthermore, the nominal group technique (NGT) was used exclusively during the stakeholder workshop to facilitate stakeholder consensus generation; as such, three of the four prioritization methods from this analysis were conducted as part of the NGT [9].

#### Prioritization method 1—Surveys with PrEP experienced HCWs

The past experience surveys were conducted with HCWs in-person, online, or over the telephone; surveys were provided online and over the phone due to the COVID-19 pandemic. Participants were asked to rate 16 PrEP delivery strategies depending on whether each strategy had been tested at their facility and the impact that each tested strategy had on improving PrEP delivery. We considered PrEP delivery to include PrEP education prior to initiation; risk screening as part of risk reduction counseling; HIV testing services; PrEP offer; PrEP counseling for initiation, adherence, and continuation; and provision of PrEP pills. Strategies were rated on a 3-point Likert scale as “Tested and improved delivery,” “Tested but did not improve delivery,” and “Did not test.”

#### Prioritization methods 2–4—Rankings from the stakeholder workshop

In the workshop, pre-small-group rankings, go-zone plot rankings, and post-small-group rankings were generated. In the pre- and post-small-group rankings, participants were asked to respond to an online REDCap survey and individually rank a set of 16 PrEP delivery strategies on their perceived effectiveness to improve PrEP delivery in MCH for pregnant women. Strategies were sequentially ranked from 1 (most effective) to 16 (least effective). The go-zone plots were generated in small groups where participants ranked each strategy on a 5-point Likert scale for perceived feasibility and effectiveness. Each small groups’ average feasibility and effectiveness score were plotted; strategies with mean feasibility and effectiveness scores of 2.5 or higher were considered within the “go-zone.”

#### Workshop evaluation

Following the workshop, participants were asked to complete a workshop evaluation survey. Participants rated their agreement with a series of statements about the workshop on a 5-point Likert scale from “Strongly disagree” to “Strongly agree.” The workshop evaluation surveys were completed online through REDCap.

#### Strategy bundling exercise

In a separate analysis, the strategy bundling exercise was tested as an alternative to concept mapping as traditional concept mapping is time-intensive and relatively technologically complex [38]. The strategy bundling exercise was conducted with HCWs through the HCW surveys. They were asked to create three combination packages using up to four strategies in each package based on which strategies they thought would work best in combination; strategies could be used in more than one bundle and some strategies were not used at all. They described their reasons for bundling certain strategies and how they envisioned the packages might be implemented.

### Data analysis

#### Strategy prioritization activities

In each of the four methods used, participants rated or ranked 16 strategies; *rating* involved assigning a value to each strategy while *ranking* involved assigning each strategy a place on a scale of one to 16 relative to one another. For this analysis, participant ratings were converted to rankings where all 16 strategies were placed on a list from 1 to 16 with 1 representing the highest aggregate rating and 16 representing the lowest. The past experience survey ratings were converted to ranks in two ways: (1) the percentage of respondents who reported the strategy as having been tested and improving PrEP delivery (versus not tested or tested and did not improve delivery) and (2) the percentage of respondents who reported having tested the strategy at all, regardless of whether or not PrEP delivery improved. The go-zone plot rankings for each strategy were calculated as an average of averages: mean feasibility and effectiveness scores from each small group were calculated, and then an overarching group average was calculated for the strategies’ overall ranks. Pre- and post-small-group rankings for each strategy were obtained by averaging the rank position across all workshop participants. After the four ranked lists were created, we used Kendall’s correlation analysis to determine the similarities between strategy prioritization profiles and provide a correlation metric between ranking profiles for each of the 17 possible methodology comparisons [39, 40]. A sensitivity analysis was also conducted, recalculating the past experience rankings to include strategies that had been tested overall rather than those that specifically tested and improved delivery. Kendall’s correlation coefficients were calculated across all relevant methodological comparisons for each of these measures. An additional sensitivity analysis, we assessed the correlation between strategies ranked in the top and bottom three positions across each method. Strategies rankings were transformed into a categorical variable (1 = top 3 strategy, 0 = middle strategy, -1 = bottom 3 strategy), and Kendall’s correlation coefficients were calculated for the six main comparisons across the four methods. Finally, the overall rankings of each method were plotted to assess ranking spread across the different ranking and rating methods.

Due to data collection errors, two strategies—“Coordination with adolescent friendly services” and “Task shifting any other component of PrEP counseling, assessment, or dispensing”—were excluded at the analysis stage from the original list of 16.

#### Strategy bundling exercise

Participants were asked to create the strategy bundles once over the phone with a study nurse and once online using a self-administered online survey; an additional sample of facility in-charges were only asked to complete the strategy bundles online. For each survey method, a composite list of all unique strategy bundles was created, and the top three and four strategy bundles were identified by calculating the frequency of respondents that selected each bundle. A sensitivity analysis was conducted to assess any differences in strategy bundle selection between phone and online survey methods among the participants who completed both surveys. Two Sankey diagrams, traditionally used to illustrate flows of energy, materials, costs, etc. between defined categories, were constructed to map the order of strategy selection in each bundle based on the first strategy selected among phone and online participants [41]; these diagrams were completed among the survey participants who selected exactly 4 strategies per bundle using SankeyMATIC [42].

#### Workshop evaluation

The proportions of participants’ agreement with each statement were graphed to show the distribution of agreement.

#### Reporting guidelines

A completed copy of the Strengthening the Reporting of Observational Studies in Epidemiology (STROBE) guidelines for cross-sectional studies can be found in Additional file 2 [43].

## Results

### Participant demographics

Out of the 185 HCWs asked to participate in the past experience surveys, 182 completed the survey (*N* = 127 for both online and phone surveys; *N* = 55 facility in-charges for online survey only). Of the 48 participants invited to the stakeholder workshop, the majority completed the pre- and post-small-group discussion rankings (*N* = 44; *N* = 40 respectively). As previously described, the PrEPARE stakeholder workshop participants were older on average than the PrEP-experienced HCWs with median age 40 and 32 respectively. Both groups had similar gender distribution and educational attainment, with 62.8% and 56.5% female, as well as 95.6% and 93.5% having attended college or university across HCWs and workshop participants respectively). Among the PrEP-experienced HCWs, there was a median of 2.3 years providing care to pregnant and postpartum women and providing PrEP to this population. Additionally, 61.8% of HCWs had received training on providing PrEP adherence counseling to pregnant and postpartum women.

### Correlation between prioritization methodologies’ ranking profiles

We compared each pair of prioritization method rankings using correlation coefficients (Table 1). The correlation was strongest and significant between the pre- and post-small-group rankings (Tau: 0.648; *p* < 0.001). There was a moderate degree of correlation trending towards statistical significance between the past-experience surveys and the post-small-group rankings (Tau: 0.385; *p* = 0.062). In a sensitivity analysis, we re-computed past experience survey rankings focusing on strategies that had been tested overall, regardless of impact on delivery (sensitivity analysis: strategy was tested at all vs original analysis: strategy was tested and improved delivery; Table 1). There was stronger correlation across all methodological comparisons; for example, comparing the past experience surveys and the post-small-group rankings, the sensitivity analysis correlation was stronger than the original (sensitivity: Tau: 0.451, *p* = 0.026 vs original: Tau: 0.385, *p* = 0.062).

**Table 1:** Kendall’s correlation coefficient analyses among four strategy prioritization methods*

	Past experience (strategies that tested and improved delivery)	Past experience (strategies tested overall)	Pre-small-group rankings	Go-zone plots (overall rank)	Go-zone plots (feasibility rank)	Go-zone plots (effectiveness Rank)	Post-small-group rankings
Past experience (strategies that tested and improved delivery)	-	-	0.429 (0.036)	0.143 (0.518)	0.231 (0.279)	0.033 (0.915)	0.385 (0.062)
Past experience (strategies tested overall)	-	-	0.495 (0.014)	0.165 (0.451)	0.253 (0.233)	0.055 (0.830)	0.451 (0.026)
Pre-small-group rankings	-	-	-	0.143 (0.518)	0.231 (0.279)	0.121 (0.591)	0.648 (< 0.001)
Go-zone plots (overall rank)	-	-	-	-	-	-	0.363 (0.079)
Go-zone plots (feasibility rank)	-	-	-	-	-	-	0.398 (0.055)^ǂ^
Go-zone plots (effectiveness rank)	-	-	-	-	-	-	0.341 (0.101)
Post-small-group rankings	-	-	-	-	-	-	-

^*^P-value is testing alternative hypothesis true Tau ≠ 0

^ǂ^Not an exact *p*-value

Additionally, in the sensitivity analysis comparing strategies ranked in the top or bottom three positions for each method, we found the highest correlation between the past experience surveys, pre-small-group rankings, and post-small-group rankings (sensitivity: Tau: 0.632 for all comparisons). In contrast, the go-zone plot rankings had lower correlation across all comparisons, ranging from a Tau of 0.298 compared to pre-small-group rankings to a Tau of 0.456 compared to the post-small-group rankings.

### Ranking spread

**Fig. 2:**
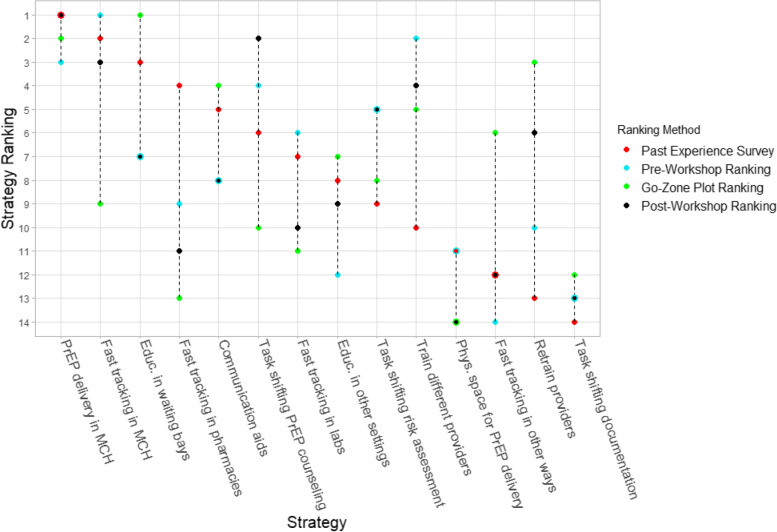
Ranking spread among past experience surveys, pre/post-small-group rankings, and go-zone plots

The ranking spread for each strategy across the past experience surveys, pre/post-small-group rankings, and go-zone plots is depicted in Fig. 2. The highest and lowest ranked strategies had the least spread with an overall difference of only two ranked positions. However, the middle ranked 12 strategies had a much higher degree of heterogeneity in the rankings across the four methodologies. Several strategies had very disparate rankings between the pre- and post-small-group ranks and the go-zone plot ranks. For example, “Task shifting PrEP counseling” was ranked 4th and 2nd in the pre- and post-small-group rankings, respectively, while it was ranked 10th in the go-zone plots. Similarly, “Fast tracking in MCH clinics” was ranked 1st and 3rd in the pre- and post-small-group rankings while it dipped to 9th in the go-zone plot rankings. When discussed in small groups, the content of the discussion for these two strategies revealed feasibility concerns. In these reports, facilitators noted broad discussions about the perceived cons of feasibility for both of the aforementioned strategies, despite the fact that there were strong feelings of perceived effectiveness for each. It was noted that both of these strategies required additional staff, may infringe on the rights of non-PrEP clients at the clinics, and may conflict with implementing partner priorities.

### Strategy bundling approaches—accuracy and pragmatic considerations

Selected strategy bundles were similar between the HCWs who completed the surveys over the phone compared to online. Among the phone surveys, 226 unique strategy bundles were identified. Of these, 179 bundles (79.2%) were selected by a single participant, 29 (12.8%) were selected by 2 participants, and 18 (8.0%) were selected by three or more participants. The top three to four strategy bundles are outlined in Table 2 and aligned most with the order in which the strategies appeared in the survey list. The strategy bundle that was most frequently identified by participants included all four strategies that related to fast tracking PrEP clients (*n* = 31; strategies 5, 6, 7, 8 in the list). Participants also identified strategy bundles that focused on task shifting in different PrEP delivery locations (*n* = 28; strategies 1, 2, 3, 4 in the list) as well as patient PrEP education and provider training (*n* = 9; strategies 9, 10, 11, 12 in the list).

**Table 2:** Most commonly identified strategy bundles from PrEP-experienced HCWs

Frequency	Phone survey (*N* = 127)	Frequency	Online survey (*N* = 182)	Frequency	Online survey among phone survey participants (*N* = 127)
28	Task shifting any PrEP counseling from nurses to HIV testing services (HTS)/HTS providers (1)	20	Fast tracking PrEP clients to reduce waiting time within MCH (5)	12	Task shifting any PrEP counseling from nurses to HIV testing services/HTS providers (1)
	Task shifting any PrEP risk assessment, including Rapid Assessment Screening Tool (RAST), from nurses to HIV testing services/HTS providers (2)		Fast tracking PrEP clients to reduce waiting time within lab (6)		Task shifting any PrEP risk assessment, including RAST, from nurses to HIV testing services/HTS providers (2)
	Task shifting documentation or data entry from nurses to a different cadre (3)		Fast tracking PrEP clients to reduce waiting time within pharmacy (7)		Task shifting documentation or data entry from nurses to a different cadre (3)
	Task shifting any other component of PrEP counseling, assessment, or dispensing (4)		Fast tracking PrEP clients in some other way (8)		Task shifting any other component of PrEP counseling, assessment, or dispensing (4)
31	Fast tracking PrEP clients to reduce waiting time within MCH (5)	20	Task shifting any PrEP counseling from nurses to HIV testing services/HTS providers (1)	12	Fast tracking PrEP clients to reduce waiting time within MCH (5)
	Fast tracking PrEP clients to reduce waiting time within lab (6)		Task shifting any PrEP risk assessment, including RAST, from nurses to HIV testing services/HTS providers (2)		Fast tracking PrEP clients to reduce waiting time within lab (6)
	Fast tracking PrEP clients to reduce waiting time within pharmacy (7)		Task shifting documentation or data entry from nurses to a different cadre (3)		Fast tracking PrEP clients to reduce waiting time within pharmacy (7)
	Fast tracking PrEP clients in some other way (8)		Task shifting any other component of PrEP counseling, assessment, or dispensing (4)		Fast tracking PrEP clients in some other way (8)
9	Delivering PrEP-related health talks in waiting bays (9)	5	Fast tracking PrEP clients to reduce waiting time within MCH (5)	4	Fast tracking PrEP clients to reduce waiting time within MCH (5)
	Conducting patient education in a different format than waiting bays (10)		Fast tracking PrEP clients to reduce waiting time within lab (6)		Fast tracking PrEP clients to reduce waiting time within lab (6)
	Retraining providers (11)		Fast tracking PrEP clients to reduce waiting time within pharmacy (7)		Fast tracking PrEP clients to reduce waiting time within pharmacy (7)
	Training different providers (12)		Delivering PrEP-related health talks in waiting bays (9)		Delivering PrEP-related health talks in waiting bays (9)
		5	Delivering PrEP-related health talks in waiting bays (9)	4	Delivering PrEP-related health talks in waiting bays (9)
			Conducting patient education in a different format than waiting bays (10)		Conducting patient education in a different format than waiting bays (10)
			Provision of communication aids (15)		Retraining providers (11)
			Coordination with adolescent friendly services (16)		Training different providers (12)

Among the online surveys, 350 unique strategy bundles were identified. Like the phone surveys, 300 bundles (85.7%) were identified by one participant, 32 (9.1%) were identified by 2 participants, and 18 (5.1%) were identified by three or more. The top four strategy bundles also focused on fast tracking (*n* = 20; strategies 5, 6, 7, 8 in the list), task shifting (*n* = 20; strategies 1, 2, 3, 4 in the list), and patient education (*n* = 5; strategies 9, 10, 15, 16 in the list). However, the online survey respondents included “provision of communication aids” and “coordination with adolescent friendly services” in the patient education bundle rather than focusing on provider training.

**Fig. 3:**
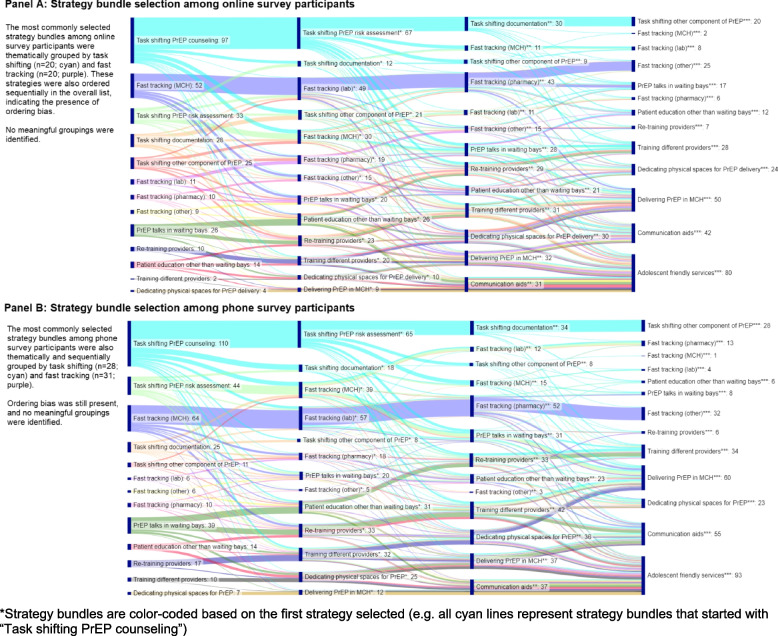
Sankey diagrams for online survey and phone survey strategy bundling exercises*. **A** Strategy bundle selection among online survey participants. **B** Strategy bundle selection among phone survey participants

The sensitivity analysis showed similar results to both the phone and online surveys (Table 2). The plurality of the most frequently identified strategy bundles consisted of consecutively ordered strategies from the overall list. The Sankey diagrams for both phone and online survey participants demonstrate the diversity of strategy bundles selected by participants (Fig. 3). The plurality of participants selected strategy bundles that were thematically grouped by task shifting (strategies 1, 2, 3, 4 in the list) and fast-tracking PrEP clients (strategies 5, 6, 7, 8 in the list). However, it is clear that certain strategies such as “Adolescent friendly services” were popular across participants of both the phone and online surveys; although this strategy was not consistently grouped with a particular strategy bundle, it was frequently included in strategy bundles (Fig. 3: panel A, *n* = 80; panel B, *n* = 93).

While completing the strategy bundling exercise, participants were given the option to explain why they selected these strategy bundles in open answer text boxes. These qualitative responses from participants highlighted a variety of motivations for strategy bundle selection, including saving time for providers and mothers, motivating clients to start and maintain PrEP use, reduce provider workload, and avoid missed opportunities to initiate clients on PrEP. Most responses focused on the impact of the individual strategies, rather than describing how the strategies might synergistically work together.

### Workshop evaluations

**Fig. 4:**
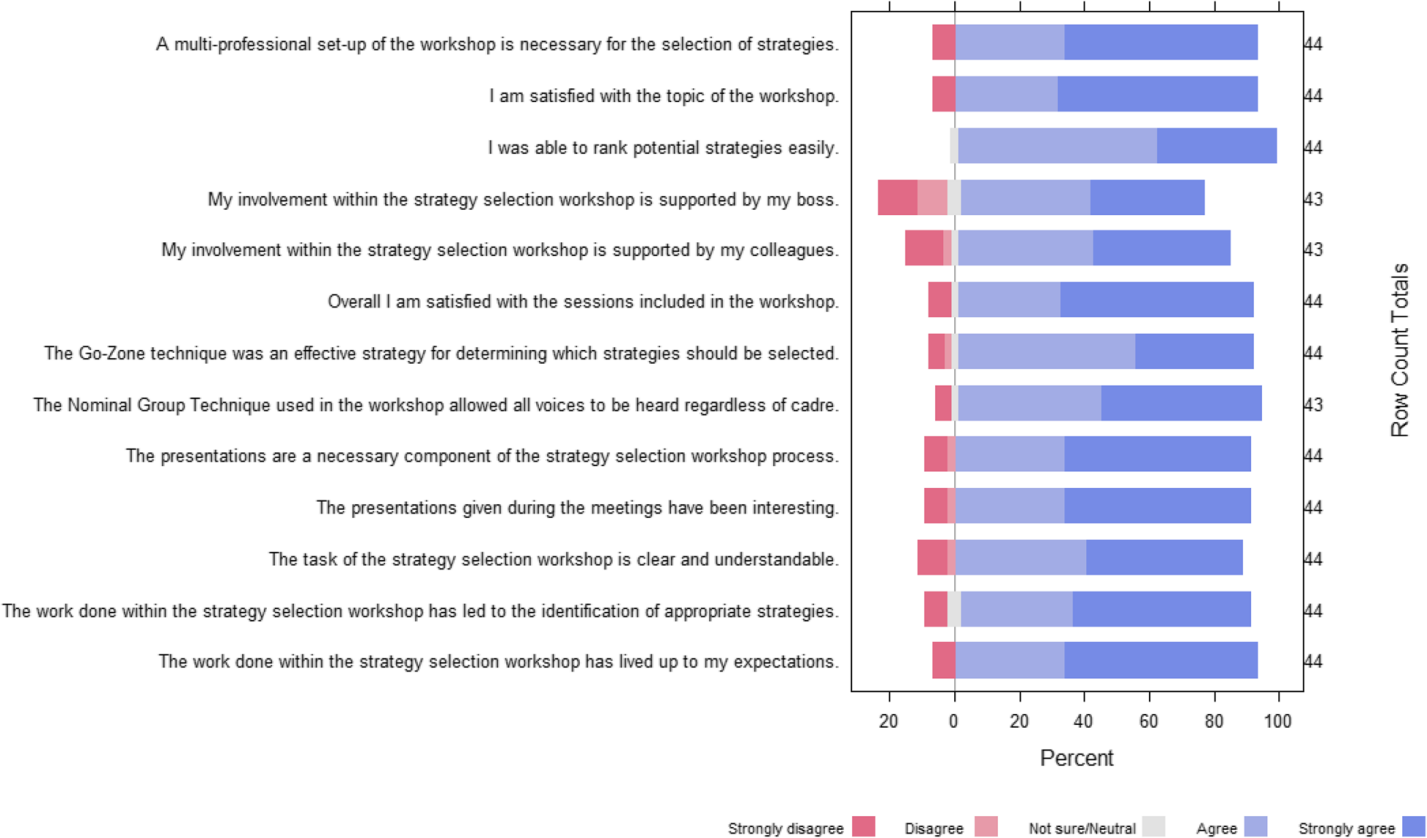
Distribution of responses to the workshop evaluation questionnaire

Of the 48 workshop participants, 46 (95.8%) completed the workshop evaluation surveys. Overall, 91% of participants agreed that there was sufficient time to discuss and review the proposed strategies, and the majority of workshop participants agreed or strongly agreed with all statements (Fig. 4). The statements with which participants most disagreed was having the support of their bosses (21%) and colleagues (14%) for participation in the workshop. No participants disagreed with the statement that they were “able to rank potential strategies easily,” and 89% of participants agreed or strongly agreed that the workshop led to the identification of appropriate strategies.

## Discussion

In this study, we compared four strategy prioritization activities with the goal of understanding the practicality of these approaches and making recommendations for streamlining strategy prioritization in future work. We observed the strongest correlation between the pre- and post-small-group rankings, suggesting that the small group discussion between the two rankings did not substantially alter individual rankings. We hypothesize several reasons why the discussions did not have an impact on individual rankings. The small groups placed participants with others of a similar cadre to reduce power imbalances and each group only discussed a subset of 3–5 of the 16 strategies to align stakeholders’ relevant experience to potential strategies. However, reviewing a subset of strategies may have made it difficult to adapt ranking of the full 16 strategies during the post-small-group rankings. Additionally, the small group rankings reflected the perceptions of those who were selected to discuss that strategy in a small group setting, preferentially those with more experience with a particular system or strategy. Finally, time constraints during the workshop may have left insufficient time for logistical processing, including adequate time for presentations on each strategy to achieve group consensus on strategy definitions. Based on these data, we recommend that future strategy prioritization workshops create and send pre-reads to participants that define the implementation science terms to be evaluated (e.g., feasibility, effectiveness) and outline the strategies to be considered. Use of pre-reads, followed by an overview presentation during the workshop, will enable a more rapid, mutual understanding of strategies and implementation science terms. Future methodologic comparisons of prioritization methods should develop a shorter list of strategies for consideration and ask participants to evaluate all potential strategies rather than a subset to learn whether small group discussions impact individual rankings.

We observed that the ranking spread across strategies using different methods were quite consistent for the highest and lowest ranked items, but less tightly aligned in the middle ranks. In our sensitivity analysis of the top and bottom ranked strategies, we observed that the past experience surveys and the pre- and post-small-group rankings were much more tightly correlated compared to the go-zone plot rankings. The correlation between methodologies may have been impacted by having too many items to rank. Ranking tasks are more difficult to complete with a larger list of items; while participants are often able to clearly identify the highest and lowest ranked items, there is often less distinction between the items in the middle of the list [44], with more unexplained variance in ranking choices as the number of ranking choices increase [45]. Strategy prioritization exercises should consider limiting the number of options when using ranking. However, in the three methods that required participants to rank all 16 strategies, there was good agreement about the top and bottom three strategies, indicating that participants were able to reach a general consensus about the strategies they would prefer to implement based on perceived feasibility and effectiveness. Had participants been required to complete the go-zone plots evaluating all 16 strategies rather than a subset, we may have observed higher correlation across these methodological comparisons. This discrepancy further demonstrates the need to rank all strategies in each method when multiple methods are being utilized.

We noted that workshop rankings correlated more closely to sensitivity analyses of past experience survey rankings focused on whether a strategy had been tested at all, rather than tested and improved delivery. Although these comparisons were drawn across two different populations, they highlight how decision inertia may impact decision-making [46, 47]. Cognitive research has shown that when new evidence is presented that aligns with an individual’s initial choice, that evidence is processed more efficiently [48]. As workshop stakeholders were familiar with which strategies had been tested rather than those that actually improved delivery, there may have been bias to align their rankings accordingly. It is particularly interesting that feasibility pre- and post-small-group rankings were more tightly correlated compared to effectiveness rankings because participants were specifically asked to consider effectiveness in their pre- and post-small-group rankings. Study staff noted that stakeholders, in particular HCWs, were more likely to view feasibility as something within their control, depending on the implementation of the individual strategy. However, effectiveness was seen as a patient-dependent construct and beyond the control of implementing HCWs, indicating that potential effectiveness may be more difficult to judge compared to feasibility. The use of pre-reads may assist in overcoming these challenges. For example, the pre-reads may be used to define implementation terms and familiarize participants with all strategies, while the workshop may be used to review the pre-read content in a presentation and conduct the ranking activities. Additionally, obtaining and presenting patient-level preferences regarding the considered strategies may provide insights further insights into strategy effectiveness; as Kenyan stakeholders perceived effectiveness to be indicative of a patient-level construct, they may benefit from understanding patient preferences as they consider strategies’ effectiveness.

Decision inertia and decision-making bias may be addressable with group decision-making or instructed dissent to encourage dissenting viewpoints [46, 49]. We utilized the nominal group technique (NGT) in this study to encourage democratic participation, as this method is designed to quickly reach consensus and has been increasingly used in medical and health services research [7, 50–54]. However, the NGT may not have been able to sufficiently overcome decision inertia because of the homogeneity of the small groups as we had created similar cadre small groups to reduce power imbalances between patients, providers, and policymakers [46]. Group strategy prioritization exercises may still be useful to interrupt decision inertia, but careful attention should be paid to balance the goals of homogeneity to reduce power dynamics and heterogeneity to interrupt decision inertia.

The results from the workshop evaluation surveys demonstrate a high level of acceptability regarding the methods for strategy prioritization utilized during the workshop. The majority of participants felt that the NGT and go-zone plots were useful to ensure that all voices were heard and to select appropriate strategies moving forward. Although the majority of participants felt there was sufficient time to discuss potential strategies, there was a slightly elevated proportion of participants who felt the tasks during the workshop were unclear; the proposed use of pre-reads may provide the additional time necessary to more fully describe the workshop activities and ensure mutual understanding of the proposed strategies among participants. Care needs to be taken to ensure that individuals’ participation is supported by their bosses and colleagues, particularly for HCWs as their absence from work may impact clinic flow; extending the workshop would likely make this stakeholder engagement strategy less acceptable.

Our strategy bundling exercise tested an approach to conducting stakeholder-engaged prioritization that required less time and resources than concept mapping. However, our strategy bundle exercise was not effective, as participants defaulted to creating bundles that mostly reflected the ordered list provided. This reflects order bias and may be partially attributable to the design of the strategy list which was compiled by broad themes (e.g., fast tracking, task shifting, etc.), prompting participants to select strategies thematically rather than creating synergistic bundles [55]. Strategy bundling exercises should consider randomizing strategy order, as is recommended in survey literature [56, 57].

In addition to the comparisons of prioritization activities, we evaluated a simplified strategy bundling exercise to determine whether this approach is comparable to more traditional approaches such as concept mapping. We compared two levels of time and staff resources by collecting strategy bundling data by phone and online survey. We found that individuals who completed the survey over the phone with study staff were much more likely to choose the required number of strategies per bundle than those who completed the survey independently online. The advantage conferred by staff correcting participants’ number of strategies selected could be overcome with online constraints that forced inclusion of the required number of strategies. Alternatively, concept mapping could be a better approach because its highly structured nature removes the possibility of incorrectly including or excluding particular strategies. A pooled analysis of 69 concept mapping studies found high internal representational validity and reliability, suggesting that concept mapping is likely a superior method of identifying strategy bundles despite its time and resource complexity [58]. We recommend that teams consider concept mapping as a first choice; if utilizing an alternative bundling approach, we recommend using surveys that enforce bundling rules. The qualitative responses participants provided explaining why they chose each strategy bundle demonstrated a variety of motivations for bundle selection, but a health systems view was not applied. Study staff noted that HCWs tended to view the potential strategies from a HCW-cost vs. client-benefit perspective, first considering the challenges to strategy implementation and weighing them against the potential benefits to clients which may explain why HCWs were more likely to highlight individual benefits to strategy implementation. Additionally, HCWs had previous experience organically testing PrEP implementation strategies at the facilities, potentially promoting the view that strategies are individual entities rather than elements of an intervention that can work in tandem to address multiple barriers. Future work in this area should consider additional prompting and targeted questions to address facility and program determinants of intervention implementation and how different types of strategies may work synergistically to produce the desired outcomes, particularly among HCWs.

Additionally, it may be difficult to achieve full consideration of strategy synergies through a strategy bundling exercise. As it remains challenging for individuals to consider and hold all potential downstream impacts of strategy combinations, previous work has called for a complex systems lens in regards to combination HIV prevention in order to differentiate between additive versus synergistic effects of multiple interventions [59, 60]. Simulation modeling-based approaches are useful for tackling complex systems in public health, including intervention evaluations prior to their actual implementation [60, 61]. Future strategy prioritization and bundling activities should consider the use of simulation modeling techniques such as discrete event simulation and system dynamics models to more accurately assess how strategy combinations will synergistically impact clinic operations and patient flow, as has been implemented in the U.S. Department of Veterans Affairs [60, 62].

### Implications for practice

As previously discussed, our recommendations for future strategy prioritization work include limiting the number of strategies for each prioritization activity and ranking all strategies in each prioritization activity when multiple activities are utilized. While the past experience surveys with healthcare workers provided a useful starting point for identifying implementation strategies that aligned with stakeholder goals, the prioritization activities from the stakeholder workshop were able to incorporate a greater diversity of perspectives and explicitly consider both feasibility and effectiveness. In the context of a stakeholder workshop, we recommend sending pre-reads to participants to explain the strategies under consideration and the implementation science terms that will be used and providing patient-level data on strategy preferences for consideration in discussions of potential effectiveness. Finally, in future strategy bundling exercises, we recommend using methods such as discrete event simulation or simulation dynamics modeling approaches to more fully account for the complexity involved in assessing strategy synergies.

### Next steps for research

Future research should directly assess the ideal number of strategies to include in prioritization activities. Additionally, there is a dearth of comparative effectiveness literature for strategy prioritization activities. There is a need for future work involving more than one prioritization method to make direct comparisons between the results of these methods in order to optimize time and resources expended on strategy prioritization.

This study included diverse stakeholder perspectives in the evaluation of modified strategy prioritization methodologies. Few studies have had the ability to draw comparisons between ranking methodologies within the same study. Several of the limitations of the PrEPARE study include potential recall bias in healthcare worker responses, power differentials in the stakeholder workshop small groups, and the use of purposive sampling. The primary limitation of this analysis is that the PrEPARE study was not designed to directly compare the strategy prioritization methodologies. However, there is still value in assessing the comparative usefulness of these methodologies as they were applied in practice and to inform future structured experiments, as has been done in studies comparing the time, resources, and yield of strategy elicitation exercises [63]. As the 16 implementation strategies were thematically identified through prior qualitative work, we were unable to systematically specify the strategies according as per Proctor et al. [64]. However, after the prioritization process, all strategies to be tested were specified by the study team. We also did not perform explicit strategy-barrier mapping, but several of the prioritized strategies matched reasonably well to the barriers identified post hoc*,* such as retraining and training new providers to address insufficient provider-patient ratios. Additionally, we were unable to analyze strategies individually to determine whether the rankings changed during the workshop due to barriers in data structure and linkages. While small group discussions may be helpful in strategy consideration, the use of go-zone plots may not be additionally useful to identify a final set of strategies for implementation. Furthermore, there was no a priori rationale for thematically grouping strategies in the provided list for the strategy bundling exercise; the challenges with strategy non-randomization presented here highlight the need for future strategy bundling activities to randomize the strategies provided to participants as part of best practices. In the past experience surveys, the use of self-reported improvements in PrEP service delivery may be subject to recall bias. However, we are utilizing PrEP-experienced HCWs’ perceived service delivery improvement to generate further discussion of potential strategies and offer a starting point for identifying implementation strategies that align with stakeholder goals. We are currently testing the prioritized strategies to provide empiric evidence of any observed improvements in PrEP service delivery.

## Conclusion

In this study, we conducted the first head-to-head comparison of pragmatic stakeholder engagement methods utilized in low-resource settings, including four implementation strategy prioritization activities and a strategy bundling exercise. We found that participants were more likely to prioritize familiar strategies and that ranking exercises were less effective with large numbers of strategies. In future strategy prioritization activities, we recommend using pre-reads to define both implementation science terminology and strategies as well as providing a short list of strategies for small group discussions, followed by a ranking of all listed strategies. Additionally, we found that a low time- and resource-intensive strategy bundling exercise was not effective in identifying strategy bundles. In future strategy bundling exercises, we recommend utilizing existing methods with high internal validity like concept mapping, or simulation modeling-based approaches.

## Supplementary Information

**Additional file 1:** Strategies prioritized for testing.

**Additional file 2:** STROBE Statement—Checklist of items that should be included in reports of cross-sectional studies.

## Data Availability

The datasets generated and/or analyzed during the current study are not publicly available due to limitations in data sharing permissions from the Kenyan ethical review boards but are available from the corresponding author on reasonable request. The datasets used and analyzed during the current study are available from the corresponding author on reasonable request.

## Abbreviations

ERC: Ethics and Research Committee
HCW: Healthcare worker
HIV: Human immunodeficiency virus
HTS: HIV testing services
IRB: Institutional Review Board
IS: Implementation Science
KNH-UoN: Kenyatta National Hospital-University of Nairobi
LMIC: Low- and middle-income countries
MCH: Maternal and child health
NGT: Nominal group technique
PrEP: Pre-exposure prophylaxis
PrEPARE: PrEP in Pregnancy, Accelerating Reach and Efficiency
RAST: Rapid Assessment Screening Tool
UW: University of Washington
WHO: World Health Organization
