# Supplementary material for: Comparison of methods to engage diverse stakeholder populations in prioritizing PrEP implementation strategies for testing in resource-limited settings: a cross-sectional study
Source: Implement Sci Commun. 2023 Jul 12;4:76. doi: 10.1186/s43058-023-00457-9 (PMC10337117; doi:10.1186/s43058-023-00457-9)
Supplement: Supplementary file 1 — Additional file 1: Strategies prioritized for testing. [file 43058_2023_457_MOESM1_ESM.docx]

| **Strategy** | **Prioritized for Testing** |
| --- | --- |
| Delivering PrEP commodities within MCH clinic instead of pharmacy | Yes |
| Fast tracking PrEP clients to reduce waiting time within MCH | Yes |
| Delivering PrEP related health talks in waiting bays | Yes |
| Fast tracking PrEP clients to reduce waiting time within pharmacy | No |
| Task shifting any PrEP counseling from nurses to HIV testing services/HTS providers | Yes |
| Provision of communication aids | No |
| Fast tracking PrEP clients to reduce waiting time within lab | No |
| Conducting patient education in a different format than waiting bays | No |
| Coordination with adolescent friendly services | Removed |
| Task shifting any PrEP risk assessment, including RAST, from nurses to HIV testing services/HTS providers | Yes |
| Training different providers | Yes |
| Dedicating certain physical space, rooms or tents, to be PrEP delivery rooms | No |
| Fast tracking PrEP clients in some other way | No |
| Retraining providers | Yes |
| Task shifting any other component of PrEP counseling, assessment, or dispensing | Removed |
| Task shifting documentation or data entry from nurses to a different cadre | No |

Additional file 1: Strategies prioritized for testing
